# GSA COVID-19 Task Force: Developing a Research Agenda

**DOI:** 10.1093/geroni/igaa057.2943

**Published:** 2020-12-16

**Authors:** Barbara Resnick, Sheryl Zimmerman

**Affiliations:** 1 University of Maryland School of Nursing, Baltimore, Maryland, United States; 2 University of North Carolina at Chapel Hill, Chapel Hill, North Carolina, United States

## Abstract

GSA COVID-19 Task Force members worked with organizational leaders and special interest groups to ascertain areas where further research about COVID-19 and older adults would be helpful. The responses were compiled into a single document to create a research agenda. Processes to share the agenda with federal agencies and partners will be shared.

